# The genus *Pseudolathra* Casey in China: new species and new records (Coleoptera, Staphylinidae, Paederinae)

**DOI:** 10.3897/zookeys.356.5979

**Published:** 2013-11-27

**Authors:** Xiao-Yan Li, Alexey Solodovnikov, Hong-Zhang Zhou

**Affiliations:** 1 Key Laboratory of Zoological Systematics and Evolution, Institute of Zoology, Chinese Academy of Sciences, 1 Beichen West Road, Chao Yang, 100101 Beijing, P.R. China; 2 Department of Entomology, Natural History Museum of Denmark/University of Copenhagen, Universitetsparken 15,Copenhagen 2100, Denmark

**Keywords:** New species, *Pseudolathra*, Paederinae, Staphylinidae, China

## Abstract

Two new species of the genus *Pseudolathra* Casey from mainland China are described and attributed to their respective species groups, *P. cylindrata* **sp. n.** from Hubei and Sichuan, and *P. superficiaria* **sp. n.** from Yunnan. *Pseudolathra pulchella* (Kraatz, 1859), *P. transversiceps* Assing, 2013 and *P. bipectinata* Assing, 2013 from Yunnan are reported from China for the first time. The history of the exploration of the Chinese fauna of *Pseudolathra* is summarized.

## Introduction

Except for the better known West Palearctic region (ten species), the taxonomy, diversity, and zoogeography of the paederine rove beetle genus *Pseudolathra* Casey, 1905 are currently somewhat unclear (Assing 2012). However, the recent revision by Assing (2012) and its supplement (Assing 2013) significantly improved the situation for the East Palearctic and Oriental regions, where the genus currently includes 19 species, some of which are distributed across both regions. Even though a thorough phylogenetic subgeneric division of *Pseudolathra* is still pending, Assing (2012) suggested that all East Palearctic and Oriental species represent a distinct lineage currently assigned to the subgenus *Allolathra* Coiffait, 1972 and subdivided into three species groups, *Pseudolathra regularis*, *Pseudolathra nigerrima* and *Pseudolathra unicolor* groups.

With respect to China, a country of vast dimensions, rich biodiversity, and with a poorly studied rove beetle fauna, the discovery of numerous new species of *Pseudolathra* has been predicted (Assing 2012, 2013). Before our study, only three species were recorded from China: *Pseudolathra unicolor* (Kraatz 1859) from Yunnan, Guangxi and Taiwan, *Pseudolathra regularis* (Sharp 1889) from Sichuan, Shaanxi, Yunnan and Jiangsu, and *Pseudolathra lineata* Herman 2003 from Jiangsu, Jiangxi and Taiwan (Assing 2012).

Based on recently collected material, two new species from mainland China are here described and illustrated: *Pseudolathra cylindrata* sp. n. from Hubei and Sichuan, and *Pseudolathra superficiaria* sp. n. from Yunnan. Three additional species, *Pseudolathra bipectinata* Assing, 2013 (Yunnan), *Pseudolathra transversiceps* Assing, 2013 (Hainan) and *Pseudolathra pulchella* (Kraatz, 1859) (Hainan) are reported from China for the first time. Using the diagnostic characters provided by Assing (2012), *Pseudolathra cylindrata* sp. n. is placed in the *Pseudolathra unicolor* species group, and *Pseudolathra superficiaria* sp. n. in the *Pseudolathra nigerrima* species group, both in the subgenus *Allolathra*. Thus, altogether eight species of *Pseudolathra* are presently known from China.

The type specimens are deposited in the Institute of Zoology, Chinese Academy of Sciences (IZCAS) and some duplicate paratypes deposited in the collection of the Natural History Museum of Denmark (Zoological Museum of the University of Copenhagen, ZMUC).

## Material and methods

Specimens were relaxed in warm water (60°C) for 10–12 hours for dissection of the last abdominal segments containing the aedeagus. The detached abdominal segments were placed into KOH (10%) for 10–24 hours (depending on the degree of sclerotization) to clean sternites VIII-IX and the aedeagus from the surrounding tissues. Then they were placed into 75 % alcohol for ca. 2 minutes and transferred to vials with glycerin for examination. After examination, the dissected parts were placed in plastic genitalia vials, which were pinned under the respective specimens. Observations, drawings and measurements were made under a compound microscope (Leica MZ–APO).

All measurements were taken with an eyepiece micrometer and are given in millimeters. Total body length was measured from anterior margin of labrum to apex of abdomen. Forebody length was measured from anterior margin of labrum to apex of elytra. All other measurements were taken and abbreviated as follows:

HL head length (from the anterior clypeal margin to the occipital constriction)

EyL eye length (in dorsal view)

AL antennal length (from the base of antennomere 1 to the apex of antennomere 11)

PL pronotum length (along midline)

EL from the apex of scutellum to the elytral posterior margin

PW pronotum width (maximal)

HW head width (including eyes)

EW maximal combined elytral width

ABW abdominal width (maximal)

## Taxonomy

### Pseudolathra cylindrata sp. n.

http://zoobank.org/4E9E7410-468B-40BA-A130-32D979426B19

http://species-id.net/wiki/Pseudolathra_cylindrata

Figure 1

#### Type material.

**Holotype**, ♂, **Hubei**, Zigui, Jiulingtou 110 m, 5.IX.1994, collected by Fasheng Li (IZCAS); **paratypes**, 1♀, same data as holotype (ZMUC); 1♀, **Sichuan**, Fengdu 200 m, 1.VI.1994, collected by Youwei Zhang (IZCAS).

#### Description.

Length: 6.5–6.9 mm; length of forebody: 3.4–3.8 mm. Body glossy, vividly colored with head capsule black, pronotum and abdomen brown, elytra black with the apical margins more or less blackish-brown; antennae and mouthparts dark reddish, legs brownish red.

Head glossy, approximately as broad as long, vertex slightly convex. Punctures on head coarse and sparse, in median dorsal portion very sparse; interstices without microsculpture, but with micropunctation. Eyes relatively small and slightly protruding laterally. HL/EyL = 2.8, eyes shorter than postocular region in dorsal view. Antennae slender, about 1.9–2.0 mm long; all antennomeres oblong.

Pronotum oblong, PL/PW = 1.2, widest at its anterior third and approximately 1.2 times as broad as head, lateral margins straight; on either side of the impunctate midline with series of 13–16 punctures, some of these punctures often accompanied by additional punctures; punctures of lateral portions sparse to moderately dense; interstices without microsculpture.

Elytra parallel-sided, EL/EW = 8.5, slightly longer than pronotum; punctures on surface arranged in 5 series in dorsal view; interstices without microsculpture. Hind wings fully developed.

Abdomen approximately as broad as elytra, wider than head or pronotum; punctation very fine and dense; interstices with microsculpture; posterior margin of tergite VII with palisade fringe.

**Figures 1.**
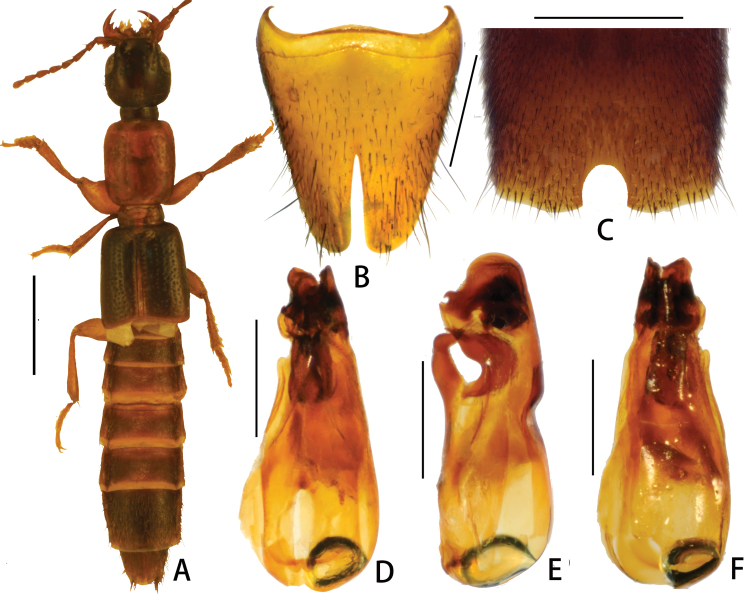
*Pseudolathra cylind rata* sp. n. from Hubei and Sichuan. **A** habitus **B** sternite VIII, male **C** sternite VII, male **D** aedeagus, dorsal view **E** aedeagus, lateral view **F** aedeagus,ventral view. Scale bars: **A** = 1 mm; **B–F** = 0.5 mm.

Aedeagus (Fig. 1D–F) about 1.25 mm long, length/width = 2.4. Dorsal plate fused with median lobe. Ventral process strongly sclerotized and curved (Fig. 1D, E). Internal sac with some strongly sclerotized structures.

Male sternite VII (Fig. 1C) with narrowly semicircular excision, margins of this excision slightly depressed; male sternite VIII (Fig. 1B) with posterior excision narrow and deep, not quite reaching middle of sternite.

Female sternites VII-VIII without any modifications.

#### Etymology.

The species name is the Latin adjective meaning cylindrical. It refers to the shape of the aedeagus of this new species.

#### Remarks.

Based on the relatively smaller body size, brownish coloration with darker head and elytra, as well as distinctly sclerotized aedeagus with an apically projecting dorsal plate, the new species belongs to the *Pseudolathra unicolor* group *sensu* Assing (2012). Within this group, the new species is externally very similar to *Pseudolathra pulchella* (Kraatz), but differs as follows: 1) the posterior excision of the male sternite VIII in the new species (Fig. 1B) is narrower and deeper (*Pseudolathra pulchella*: figure 39 in Assing 2012); 2) the aedeagus in *Pseudolathra cylindrata* sp. n. is of a different shape, with its ventral process forming a distinct perforation in lateral view (Fig. 1D–F; *Pseudolathra pulchella*: see figures 33–34 in Assing 2012).

#### Distribution.

*Pseudolathra cylindrata* sp. n. is known only from the type locality: Jiulingtou in Zigui County, Hubei. The type series was collected in the period from June to September, the altitudes ranging from 100 to 200 m.

### Pseudolathra superficiaria sp. n.

http://zoobank.org/32416B79-5297-4242-91CD-CE76BDCE00B2

http://species-id.net/wiki/Pseudolathra_superficiaria

Figure 2

#### Type material.

**Holotype**, ♂, **Yunnan**, Mengla County, Township Yaoqu, 1030 m (21.73°N, 101.52°E), 4.X.2010, leg. Xi Zhang (IZCAS).

#### Description.

Length: 6.5 mm; length of forebody: 3.2 mm. Body black, glossy; abdomen with posterior and lateral margins slightly dark reddish; legs, antennae and mouthparts reddish.

**Figures 2.**
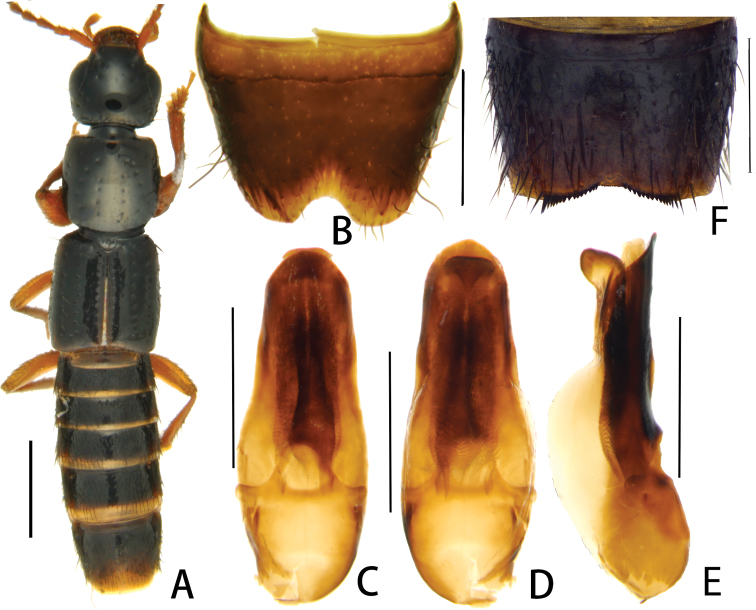
*Pseudolathra superficiaria* sp. n. from Yunnan. **A** habitus **B** sternite VIII, male **C** aedeagus, ventral view **D** aedeagus, lateral view **E** aedeagus, dorsal view **F** sternite VII, male. Scale bars: **A** = 1 mm; **B–F** = 0.5 mm.

Head (Fig. 2A) weakly transverse, approximately 1.1 times as wide as long; vertex slightly convex, posterior angles marked. Median area almost impunctate, lateral portions with coarse and very sparse punctures; punctures around eyes and along neck relatively fine and dense; interstices without microsculpture and micropunctation. Eyes large and bulging, HL/EyL = 1.7, approximately 1.5 times as long as postocular region in dorsal view. Antennae slender, about 1.85 mm long; antennomeres III-X with very narrow bases and broadened apices.

Pronotum (Fig. 2A) approximately as long as broad and as wide as head, anterior angles distinct and posterior angles round; on either side of the impunctate midline with series of 1+4 coarse punctures in dorsal view; lateral portions with very sparse and coarse punctures; some of those coarse punctures having additional smaller punctures.

Elytra with EL/EW = 8.7, slightly longer than pronotum, with fine epipleural ridge; punctures on dorsal surface arranged in 3 pronounced series on each elytron; interstices without microsculpture. Hind wings fully developed.

Abdomen approximately as broad as head or pronotum, but narrower than elytra; punctation on tergites III-V very coarse and dense, tergites VI-VIII with punctures relatively fine and dense; interstices without microsculpture; posterior margin of tergite VII with palisade fringe.

Aedeagus about 1.1 mm long, length/width = 2.7, weakly sclerotized, shaped as in Fig. 2C–E.

Malesternite VII (Fig. 2F) with posterior margin weakly and broadly concave, on either side of the middle with comb of stout, black, spine-like setae increasing in length and thickness laterad, margins of the concavity slightly depressed and glabrous; male sternite VIII (Fig. 2B) with semicircular, broad and relatively shallow posterior excision.

Females unknown.

#### Etymology.

The species name is the Latin adjective meaning superficial. It refers to the shallow posterior excision of the male sternite VIII of the new species.

#### Remarks.

Based the synapomorphic modifications of the male sternite VII (posterior margin bipectinate and notched in the middle) and the similar morphology of the aedeagus, the new species is closely allied to *Pseudolathra bipectinata*. For illustrations of *Pseudolathra bipectinata* see figures 17–18, 22–23 in Assing (2013). Like *Pseudolathra bipectinata*, *Pseudolathra superficiaria* belongs to the *Pseudolathra nigerrima* group and can easily be distinguished from other species of this group by the broad and shallow (not deep and narrow) excision of the male sternite VIII, and by the characteristic structure of the aedeagus.

In fact, the shallow and broad excision of the male sternite VIII was previously reported as a uniquecharacter of the monotypical *Pseudolathra regularis* group (Assing 2012), however, the exact shape of this shallow excision is different in both species. Besides, *Pseudolathra superficiaria* sp. n. differs from *Pseudolathra regularis* by the more transverse head with larger eyes, the much more transverse pronotum, and the shape of the aedeagus.

#### Distribution.

The only known specimen of *Pseudolathra superficiaria* sp. n. was found in the leaf litter of the forest of rubber trees near the center of Yaoqu, a town in Mengla County, Yunnan. It was collected in October by sifting leaf litter at an altitude of 1030 m.

### Records of the genus *Pseudolathra* species from China

#### Pseudolathra bipectinata

Assing, 2013, first record for the territory of China

http://species-id.net/wiki/Pseudolathra_bipectinata

##### Material examined.

4♂♂, 3♀♀, **Yunnan**, County Jinghong, Nabanhe Nature Reserves 730 m, 16.IV. 2009, leg. Lingzeng Meng; 1♂, 3♀♀, same data but 770 m, 6.IV.2009; 2♂♂, 3♀♀, same data but 710 m; 2♂♂, 3♀♀, same data but 730 m, 6.VI.2009; 3♂♂, 2♀♀, same data but 760 m, 6.IV.2009; 1♂, 1♀, same data but 770 m; 2♂♂, 3♀♀, same data but 800 m, 16.IV.2009; 3♂♂, 3♀♀, same data but 1110 m, 16.V.2009 (IZCAS, ZMUC).

##### Comments.

*Pseudolathra bipectinata* is illustrated and its distribution mapped in Assing (2013: figures 17–23, map 3). It was previously known only from Laos. The above specimens collected at altitudes between 200 and ca. 1000 m in southern China represent a new county record.

#### Pseudolathra lineata

Herman, 2003

http://species-id.net/wiki/Pseudolathra_lineata

##### Specimens examined.

1♀, 1♂, **Sichuan**, City Luzhou, 4.VI.1974, leg. Yinheng Han (IZCAS).

##### Comments.

The previously known distribution of *Pseudolathra lineata* Herman included Japan, as well as Taiwan and the mainland Chinese province, Jiangsu (Assing 2012). The above record from Sichuan represents a new province record.

#### Pseudolathra pulchella

(Kraatz, 1859)

http://species-id.net/wiki/Pseudolathra_pulchella

##### Material examined.

**Hainan**, 3♂♂, 5♀♀, Qiongzhong City, Limu Shan 600 m, 15.V.2007, leg. Zongyi Zhao; 1♀, Lishui, Diaoluo Shan (light trap, 18.66N, 109.93E) 60 m, 24.III.2007, leg. Hongliang Shi and Feng Yuan; 3♂♂, 2♀♀, **Guangdong**, Cheba Shan, Nature Reserve Conservation Zone, 370 m, 22-26.VII.2008, leg. Zhuo Yang (IZCAS, ZMUC).

##### Comments.

The morphology of *Pseudolathra pulchella* was redescribed by Assing (2012); an updated distribution map was provided by Assing (2013). The above specimens represent new province records from Hainan and Guangdong.

#### Pseudolathra regularis

(Sharp, 1889)

http://species-id.net/wiki/Pseudolathra_regularis

##### Specimens examined.

3♀♀, 2♂♂, **Beijing**, Haidian, Yingtaogou, 19.V.1997, leg. Haisheng Zhou; 1♂, Changping, 20.X. 1988, leg. Huiying Wang; 1♀, Haidian, Qionglongqiao, 15.V.1997, leg. Haisheng Zhou; 1♀, Haidian, Jiufeng, 3.VII.1997, leg. Haisheng Zhou; 1♀, Haidian, Anheqiao, 13.VI.1996, leg. Haisheng Zhou (IZCAS, ZMUC).

##### Comments.

Previously, this species was known from Japan, and from the southern and western parts of China (Jiangsu, Yunnan, Shaanxi, and Sichuan) (Assing 2012, 2013). The above material expands the known distribution of *Pseudolathra regularis* to northern China.

#### Pseudolathra transversiceps

Assing, 2013

http://species-id.net/wiki/Pseudolathra_transversiceps

##### Specimens examined.

♂, **Hainan**, Bawang Shan, 1000 m, 28.XI.2009, leg. Zongyi Zhao; ♂, **Yunnan**, City Jinghong, County Menghai, Nabanhe nature reserve conservation zone, 1110 m, 16.III.2009, leg. Lingzeng Meng (IZCAS, ZMUC).

##### Comments.

*Pseudolathra transversiceps* was previously known only from Vietnam (Assing 2013, map 4). The examined specimens from southern China, which were collected at altitudes ranging from 400 to 1000 m, expand the known distribution northwards.

#### Pseudolathra unicolor

(Kraatz, 1859)

http://species-id.net/wiki/Pseudolathra_unicolor

##### Specimens examined.

3♀♀, **Yunnan**, Xishuangbanna Yaoqu, 5.VIII.2006, leg. Gang Yao; 1♂, Xishuangbanna Xiaomengyang 850 m, 20.VIII.1958, leg. Yiran Zhang; 2♀♀, Luxi, 24.VI.1958; 2♀♀, **Jiangxi**, Dongxiang, 6.VII.1957, leg. Xuewen Ying; 1♀, Huichang, 20.VI.1959; 1♀, **Fujian**, Shaowu, 1980, leg. Zhanghong Qiu; 1♀, **Guangxi**, Guilin, Yan Shan, 12.V.1963, leg. Chunguang Wang; 1♀, Guangdong, Boluo Xiangshui, 30.V.1965, leg. Youwei Zhang; 6♀♀, 2♂♂, **Tonkin**, Hoa–Binh, 1940, leg. A. de Cooman (IZCAS, ZMUC).

##### Comments.

*Pseudolathra unicolor* is a widespread species, its distribution ranging from the Himalaya deep into the Oriental region (Assing 2012, 2013). The above mentioned material indicates that it is common and widespread in southern China (Jiangxi, Guangxi and Fujian).

## Supplementary Material

XML Treatment for Pseudolathra cylindrata

XML Treatment for Pseudolathra superficiaria

XML Treatment for Pseudolathra bipectinata

XML Treatment for Pseudolathra lineata

XML Treatment for Pseudolathra pulchella

XML Treatment for Pseudolathra regularis

XML Treatment for Pseudolathra transversiceps

XML Treatment for Pseudolathra unicolor

## Appendix

Cover letter.
