# Supplementary material for: The genus Pseudolathra Casey in China: new species and new records (Coleoptera, Staphylinidae, Paederinae)
Source: Zookeys. 2013 Nov 27;(356):1–9. doi: 10.3897/zookeys.356.5979 (PMC3867107; doi:10.3897/zookeys.356.5979)
Supplement: Supplementary file 9 — Cover letter. [file ZooKeys-356-001-s001.doc]

Dear Editor:

I’m enclosing here with a manuscript entitled “**The genus *Pseudolathra* Casey in China: new species and new records (Coleoptera: Staphylinidae: Paederinae)**”. We would like to submit this *Original Article* for publication in “*ZooKeys***”.**

With the submission of this manuscript we would like to inform you that the above mentioned manuscript is a new result of our study and submitted only to your journal. We affirm that the manuscript has been prepared in accordance with *ICZN* and *ZooKeys* guide for authors. All authors have approved the manuscript and agreed with submission to *ZooKeys*. The submitted manuscript is research article.

We are very pleased to know any suggestions to improve the manuscript. Thanks you very much for consideration.

Best regards,

Xiaoyan Li

Alexey Solodovnikov

Hongzhang Zhou
